# Minimally invasive technique for gastric GIST at challenging locations: single incision surgical gastroscopy

**DOI:** 10.1007/s13304-023-01484-w

**Published:** 2023-04-01

**Authors:** Jurrien Stiekema, Joanna Luttikhold, David Heineman, Maarten Neerincx, Freek Daams

**Affiliations:** 1 Department of Surgery, Amsterdam UMC Location VUmc, Amsterdam, The Netherlands; 2 Department of Gastroenterology and Hepatology, Amsterdam UMC Location VUmc, Amsterdam, The Netherlands

**Keywords:** Gastrointestinal stromal tumor, Stomach, Surgery, Laparoscopy

## Abstract

Organ sparing resection of gastrointestinal stromal tumors (GISTs) located in the proximal stomach or esophagogastric junction can be challenging, resulting in proximal or total gastrectomy to facilitate a radical resection without tumor spill. We developed and evaluated a single incision surgical gastroscopy (SISG) procedure to provide a technically feasible alternative for the removal of gastric GISTs at these challenging locations. We developed an endoluminal resection of gastric GISTs through a small single abdominal incision and longitudinal ventral gastrotomy. Patients with a proximal tumor location, in whom a wedge resection was deemed challenging on pre-operative investigation were included in the current series. Safety, short-term oncological and surgical outcome were evaluated. We performed SISG in six consecutive patients with histopathological proven or suspected gastric GIST. In all patients, the procedure was performed successfully with no tumor rupture. The mean operative time was 61 min and there were no significant complications. Pathological examination showed a microscopically radical resection in all patients. Single incision surgical gastroscopy is a feasible technique with excellent short-term oncological and surgical outcomes. This technique serves as a good alternative for complicated resections for gastric GISTs at challenging locations.

## Introduction

The majority of gastrointestinal stromal tumors (GIST) is found in the stomach (60–70%) and small bowel (20–25%) [1]. In patients with localized disease, radical surgical resection is the primary treatment modality. For patients with small tumors located in the stomach, surgical treatment is mostly limited to a relatively simple gastric wedge resection since there is no need for wide resection margins and a lymphadenectomy is not routinely performed. Intra-operative tumor spill, however, must be prevented since it is associated with a worse outcome [2]. For GIST located close to the esophagogastric junction (EGJ), surgical resection can be challenging, as it requires extensive mobilization of the EGJ and carries the risk of post-operative esophageal stricture. It often requires a proximal or total gastrectomy to facilitate adequate resection. In more recent years, alternative strategies including endoscopic resection for smaller GISTs and laparoscopic transgastric approaches have been developed to facilitate organ-sparing surgery [3]. In this paper, we describe the safety and outcome of a new single incision surgical gastroscopy (SISG) technique for the treatment of small-to-medium-sized GISTs located in the proximal stomach, which can overcome the abovementioned difficulties.

## Methods

### Patients

All patients who underwent resection of a suspected gastric GIST using this technique in 2017, 2018, and 2019 were prospectively included. Standard diagnostic work-up involved endoscopy with tumor biopsy and computed tomography scan (CT scan) of the thorax and abdomen. Endoscopic ultrasonography and fluorodeoxyglucose positron emission tomography (FDG-PET) were performed on indication (i.e., for additional tumor samples and to rule out metastatic disease). Only patients with localized disease, preferably located in the proximal stomach (i.e., cardia/fundus) and an intraluminal tumor location within the gastric wall, were eligible for resection using SISG. Moreover, all patients were discussed in our multidisciplinary team meeting pre-*** and postoperatively. The Internal Ethics Committee approved this study and all patients gave informed consent.

### Surgical technique

**Fig. 1:**
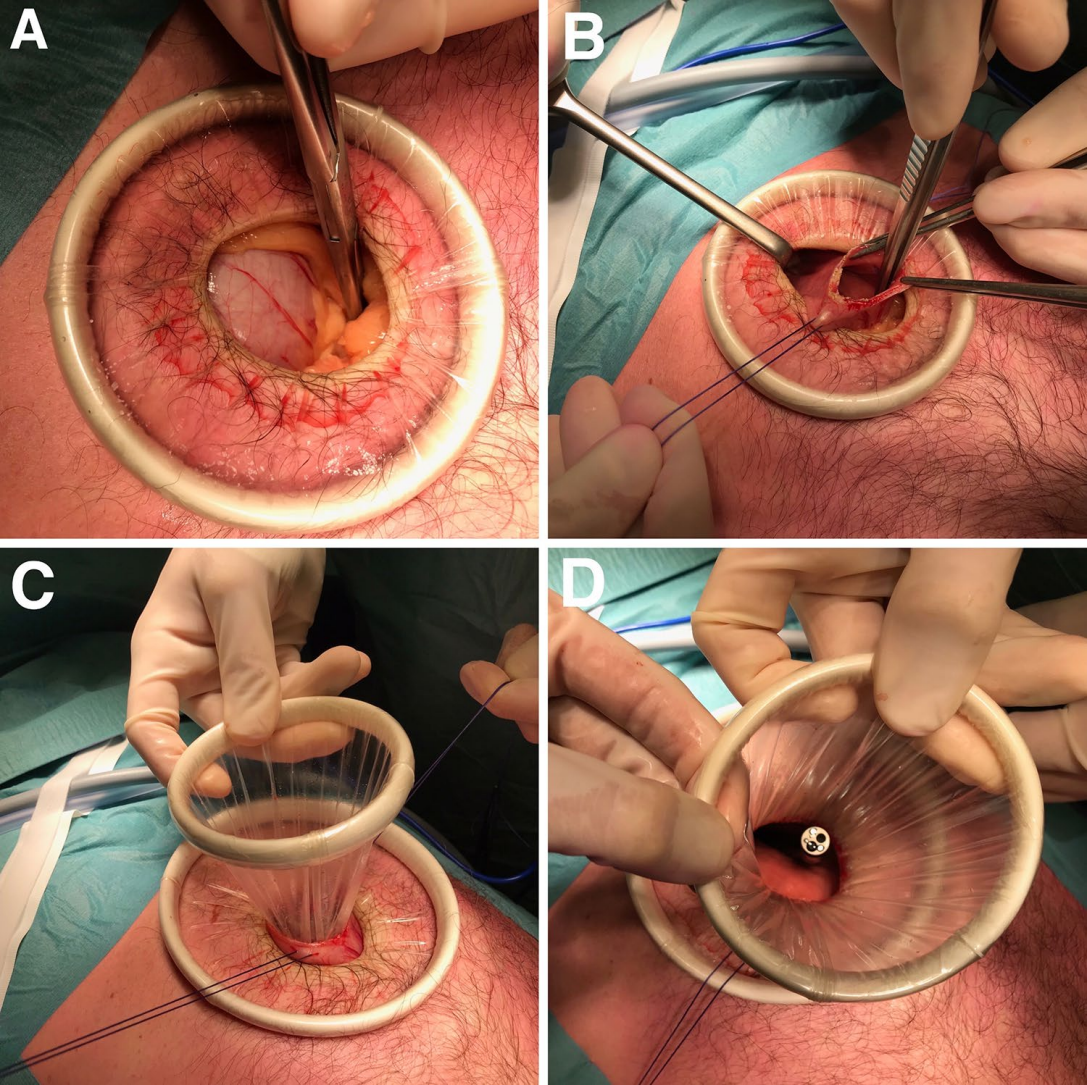
Laparotomy and gastric entry. **A** Upper abdominal midline laparotomy and introduction of the medium size Alexis wound protector^®^. Identification of the stomach. **B** Incision in anterior gastric wall with corner sutures for additional support. **C** Introduction of the small size Alexis wound protector^®^ in the stomach. **D** View of the orally introduced endoscope

**Fig. 2:**
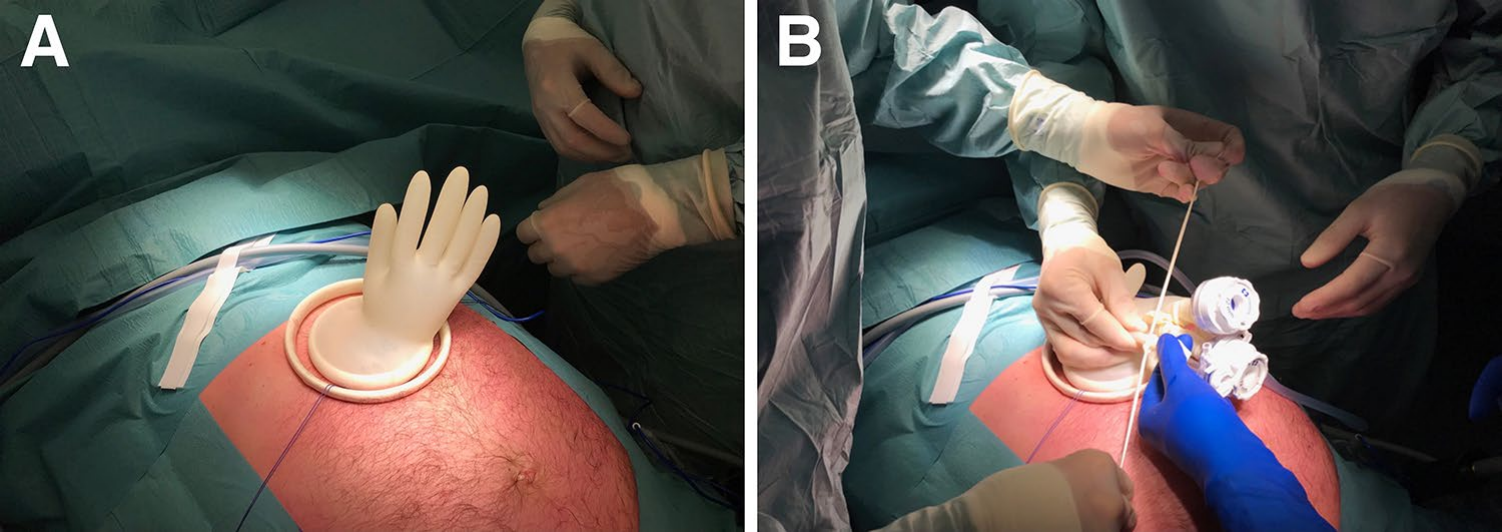
Gastric insufflation and trocar introduction. **A** Enabling gastric insufflation by wrapping a surgical glove in the Alexis wound protector^®^. **B** Trocars are introduced through the glove fingers and secured air tight using a finger cut from a second surgical glove

**Fig. 3:**
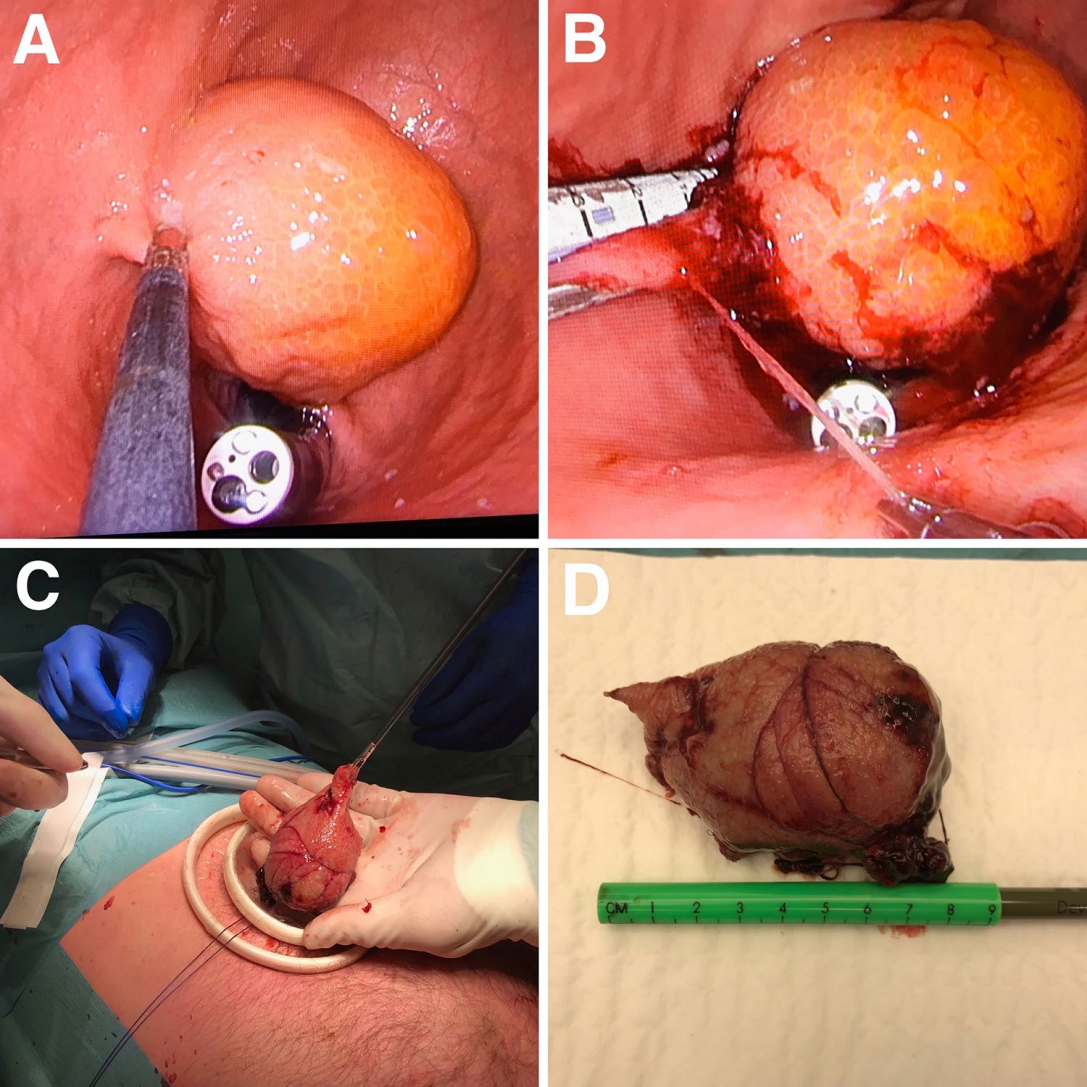
Intra-gastric resection and pathology specimen. **A** Intra-gastric view of the tumor located in the cardia with the endoscope passing the gastro-esophageal junction. **B** Positioning of the stapler device. In this case, a mucosal traction suture is used. **C** Extraction of the resection specimen. **D** The resection specimen with a ruler for size

For this procedure, general anesthesia is used and the patient is placed in supine, split-leg position (French position). Standard antibiotic prophylactics are used. The surgeon stands between the patient’s legs with the assistant and the scrub nurse on the left and right side of the patient. A mini laparotomy is performed using a 4-cm subxiphoidal midline incision. Next, a dual ring wound protector is placed and the stomach is identified (Fig. 1a). The stomach is insufflated by endoscopic gastroscopy or nasogastric tube (Fig. 1d). A 3-cm gastric longitudinal incision is made over the antrum and corner sutures are placed at both ends (Fig. 1b). Next, either a single incision laparoscopic surgery (SILS)-port or a second wound protector with a surgical glove placed over the outer woundprotector ring can be used (Fig. 1c, 2a), facilitating gastric insufflation and placement of laparoscopic trocars (Fig. 2b). After its identification, the tumor is lifted to enable placement of the endoscopic stapling device (36 to 41 mm staple height). The endoscope or nasogastric tube is used as a bougie to prevent narrowing of the EGJ. A mucosal traction suture can be used to ensure adequate stapler positioning (Fig. 3a, b). In case of staple line bleeding, hemostasis can be performed using metal clips. The resection specimen is extracted through the double wound protector, inspected for macroscopic radicality and sent to the pathology department (Fig. 3c, d). The gastric wound protector can then be removed. The stomach incision is closed with a running, double layered, inverting suture.

### Outcomes

Post-operative complications were graded according to the five-point scale as proposed by Dindo and Clavien. Post-operative mortality was defined as in-hospital death and/or within 30 days after surgery. Tumor-free resection margins were classified as R0 resection. A microscopic non-radical resection was defined as R1 resection and a macroscopic non-radical resection was defined as R2 resection. Adjuvant treatment was installed according to the advice of the post-operative multidisciplinary team meeting.

## Results

In the study period, we performed the single incision surgical gastroscopy technique in six patients with a suspected GIST. Patient, tumor, and surgical characteristics are shown in Table 1. All patients had localized disease and all tumors were proximally located in the gastric cardia or fundus. In four patients, GIST diagnosis was histologically confirmed in endoscopic biopsies, whereas in two patients, histopathology was inconclusive. In these two patients, the endoscopic and radiologic tumor aspect was highly suspected for GIST, and therefore resection was planned. Oral intake was resumed on the first post-operative day. Besides some mild complaints of nausea, all patients had an uncomplicated recovery and were discharged between POD 2–4. Histopathology results showed a radical R0 resection in all patients and a tumor size between 20 and 90 mm. Intra-operative tumor rupture did not occur in any of the patients. In one patient, histopathology was not entirely conclusive but favored a leiomyoma as the tumor was immunohistochemically positive for CD117 as well as for desmin. The risk of progressive disease according to risk classification by Miettinen et al [4]. was either very low (*N* = 2), low (*N* = 2), or moderate (*N* = 2) None of the patients received adjuvant therapy.

**Table 1:** Patient, tumor and surgical characteristics

Case	Sex	Age	Tumor location	Tumor size	Operative time	Radicality	Mitotic rate	Complications
A	M	72	Cardia	52 mm	93 min	R0	0/50 HPF	None
B	F	68	Cardia	20 mm	53 min	R0	0/50 HPF	None
C	F	68	Fundus	25 mm	42 min	R0	6/50 HPF	None
D	M	73	Fundus	42 mm	46 min	R0	3/50 HFP	None
E	F	67	Fundus	25 mm	72 min	R0	11/50 HPF	None
F	M	63	Fundus	90 mm	66 min	R0	5/50 HPF	None

*R0*: microscopically radical, *HPF*: high power

## Discussion

In this report, a SISG technique to remove gastric GISTs at challenging locations is described. In a small prospective case series, the feasibility of this technique without compromising the oncological safety was shown. In the recently updated European Society for Medical Oncology (ESMO) guidelines, the treatment of localized GIST is primary surgical resection if a radical R0 resection seems feasible [5]. Reported survival rates for small, low-risk GISTs are generally favorable after an R0 resection [6–8], and the introduction of imatinib has significantly improved the prognosis of patients with larger, high-risk GISTs [9]. More recently, laparoscopic techniques have been introduced for the treatment of GISTs. Although no large randomized controlled trials have been performed comparing open versus laparoscopic surgery, results from comparative case series show similar oncologic results with improved short-term outcome with laparoscopic surgery [10, 11]. In these series, the R0 resection rate, as well as the rate of tumor rupture were comparable. Especially the latter has been associated with poor prognosis in several studies [7, 9]. In a recent study by Hølmebakk et al., the 5-year recurrence free survival for patient with tumor rupture was 35%, compared to 88% in patient without tumor rupture. In a multivariate analysis including mitotic index, tumor size, and resection margin, tumor rupture was independently associated with recurrence, further emphasizing the need for meticulous surgery [3]. The general consensus is that iatrogenic tumor rupture during GIST surgery is a catastrophic event which must be prevented. Consequently, the current ESMO guidelines discourage laparoscopic resection of larger GISTs because of the possible risk on tumor rupture [5].

Nonetheless, due to the promising results of minimally invasive surgery for GISTs, a laparoscopic wedge resection has become a widely accepted procedure for localized gastric GISTs. A retrospective study by Khoo et al. showed that a laparoscopic wedge resection is also feasible and safe for larger (> 5 cm) gastric GISTs, with similar oncologic outcomes compared to open wedge resection [12] However, this technique, especially for large tumors, is accompanied by resection of a significant amount of healthy tissue. While in GISTs at the EGJ, this should be avoided since it could lead to strictures of even (proximal) gastric resections. In patients with tumors located on the dorsal wall of the stomach, De Vogelaere et al. used a laparoscopic approach with an anterior gastrotomy and tumor removal by placing an endoscopic stapler at the base of the tumor [13] Although no iatrogenic tumor rupture was described, extracting the specimen laparoscopically via a gastrotomy still constitutes a risk of intraperitoneal tumor spill, especially in larger tumors with ulceration or bleeding.

For smaller GISTs, several different endoscopic techniques have been described in recent literature [14] . One concern with endoscopic removal is the occurrence of a perforation of the gastric wall during the procedure and the risk on intra-abdominal tumor spill. In a recent study by Song et al., 75 of 195 (38%) patients who underwent an endoscopic resection of a gastric GIST had a perforation of the gastric wall during the procedure [15]. In 27 patients, tumor rupture occurred during the procedure. None of these patients developed disease recurrence, including 14 patients who also had a perforation of the stomach wall during the procedure. One possible explanation could be that all these ruptures occurred largely intraluminal. In a recent overview article aimed at defining tumor rupture, intraluminal tumor perforation was not considered tumor rupture since the survival of patients with this event was not different from patient with intact tumor resection [16]. Another explanation could be the selection of small, low-risk GISTs in this study (median size 25 mm, range 8–50 mm).

One of the first reports on laparoscopic intra-gastric surgery is by Ohashi, and describes a technique to remove early gastric adenocarcinoma by directly placing trocar ports through the abdominal and gastric wall into the gastric lumen [17]. Tumor removal is performed using submucosal dissection. In later series, a similar approach is described for the resection of submucosal tumors, including GISTs [18–20]. In one of the larger case series, Kanehira et al. present the short- and long-term result after removal of GISTs at the EGJ using intra-gastric surgery [21]. Both short- as well as long-term oncological outcomes are excellent and post-operative morbidity is limited. The authors propagate the use of submucosal dissection, given the location at the EGJ, facilitating a more precise resection compared to a stapling technique. Using the endoscope or nasogastric tube in the current study, the patency of the EGJ was not compromised.

In our experience, the SISG technique described in the current paper is a useful technique for gastric GISTs located at challenging locations. The gastric insufflation gives an excellent overview of the exact tumor location and provides sufficient working space for working with laparoscopic instruments directly in the stomach. This is an advantage compared to earlier described combined laparoscopic/endoscopic techniques using endoscopic mucosal dissection for tumor removal [22]. As a result, this technique can also be executed safely on large and high-risk GISTs unsuitable for endoscopic resection in limited operating time. The use of staplers negates the risk of gastric wall perforation during resection. There is no need for mobilization of the EGJ, which reduces the risk of esophageal stricture or tumor rupture. The use of wound protectors to facilitate a direct, single incision access into the stomach prevents intra-abdominal tumor spill during the procedure and at tumor extraction compared to earlier described laparoscopic techniques.
